# Chronic Albuminuria Treated with Iodide of Potassium

**Published:** 1876

**Authors:** T. S. Sharpe

**Affiliations:** Natches, Mississippi


### FIVE CASES OF CHRONIC ALBUMINURIA TREATED SUCCESSFULLY WITH LARGE DOSES OF IODIDE OF POTASSIUM.

By T. S. Sharpe, M.D., of Natches, Mississippi.

Case I. H. M—, mechanic, æt. 80, was seen in summer of 1873. Was feeble and emaciated, appetite good, digestion unimpaired; bowels constipated, face puffy, had been so for a month. Urine highly albuminous.

He was treated with iodide of potassium, 10 grs. three times daily. At the end of seven days the dose was increased to 15 grs. and afterwards gradually until he took ½ drachm morning, noon and night, when the puffiness of the face disappeared and the albumen gradually lessened in quantity and disappeared. He was dismissed in forty days.

Case II. In January, 1874, J. H——, engineer, æt. 28, consulted me. His urine was albuminous, and contained epithelial casts and cells and some blood. He began the use of the potassium in five grain doses, but was not benefitted until he increased the quantity to 22 grs. Albumen, cells and casts then gradually disappeared. To arrest the escape of blood corpuscles, I gave him 10 gr. doses of galic acid. He was dismissed in thirty-two days. At one stage of the disease this patient urinated 17 times in 24 hours.

CASE III. S. B——, planter, æt. 65, consulted me for "general debility, slow fever, and dropsy" in February, 1874. I found a very large quantity of albumen in his urine. He passed only a small quantity of urine at a time, but as often as every two hours night and day. Under the iodide of potassium treatment and a ferruginous tonic he was restored to health in forty-four days.

CASE IV. E. B——, negro, æt. 58, came "to be cured of bloody water and a stricture." Was much emaciated; had been under treatment for five months. Urinated every two hours, and the violent straining which accompanied each act forced feces from his bowels. The urine not only contained blood, but albumen in considerable quantity. Dilatation of the urthral strictures gave him great relief. He soon passed urine without an effort, which enabled the sphincter to control the rectum. Iodide of potassium was given in increasing doses until he took 32 grs. morning, noon and night. On the thirty-fourth day from the initial visit all traces of albumen had disappeared. Galic acid arrested the escape of blood.

CASE V. A young woman, æt. 22, dyspeptic and dropsical. Her urine was albuminous, and contained epithelial casts and cells. Until relieved of dyspepsia her stomach could not tolerate the potassium. On the 12th day, however, a tolerance was established, and the salt was administered, and the quantity cautiously increased, until she took 36 grs. daily, when the symptoms of chronic albuminuria disappeared.

*Remarks.*—In none of these cases did the iodide produce the peculiar unpleasant results attributed to large doses of the remedy, but it invariably increased the appetite and the flow of urine.

I questioned each patient as to the probabilities of syphilitic taint being present: every one protested against the suspicion.

My friend, Dr. John C. Inge, of this, place, has treated, within the past two years, four cases of this disease with large doses of the iodide of potassium, with similar results.—*American Journal of the Medical Sciences.*

